# Gas Sensor Based on Highly Effective Slot-Die Printed PEDOT:PSS@ZnO Hybrid Nanocomposite for Methanol Detection

**DOI:** 10.1021/acsami.4c03131

**Published:** 2024-06-04

**Authors:** Talitha Ramos Canabarra dos Santos, Maiara de Jesus Bassi, Morgana Muller de França, Júlia Ketzer Majewski, Marcos Vinícius Woiski Barcote, Anne Elize Puppi Stanislawczuk, Lucimara Stolz Roman

**Affiliations:** † DiNE - Nanostructured Devices Laboratory at Physics Department, Federal University of Paraná, 81531-980 Curitiba, Brazil; ‡ PIPE - Graduate Program in Materials Science and Engineering, Federal University of Paraná, 81531-980 Curitiba, Brazil; § Next Chemical, João Chede, 2245, 81170-220 Curitiba, Brazil

**Keywords:** gas sensor, methanol, slot-die, flexible substrate, hybrid active layer, PEDOT:PSS@ZnO

## Abstract

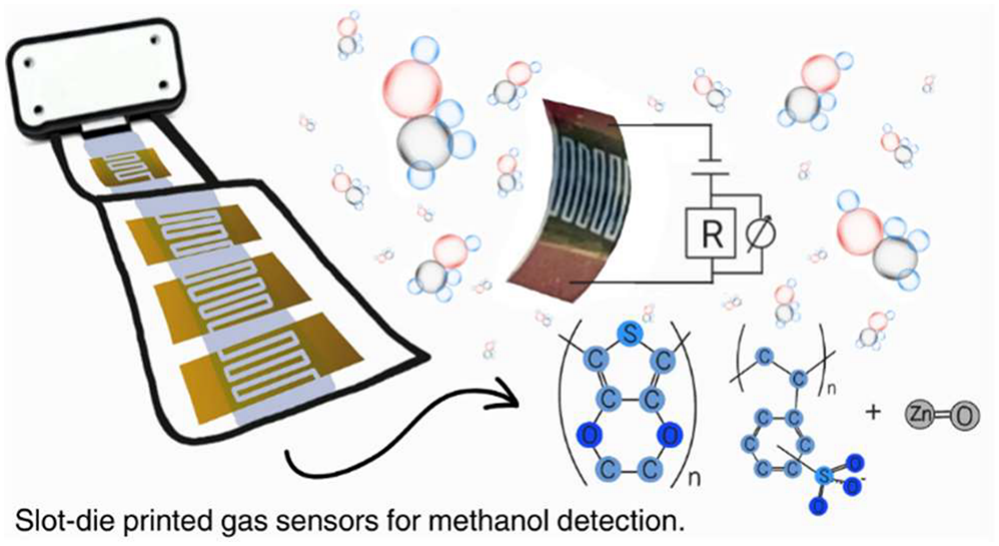

This study presents the development of gas sensors based on the PEDOT:PSS@ZnO hybrid active layer slot-die printing aqueous ink. Two different zinc oxide (ZnO) nanoparticles were studied to form the nanocomposites, as well as the use of glass and PET substrates to manufacture the devices. Despite the influence of the morphology of the active layer, all device variations studied here exhibited high response values for methanol gas at room temperature, in addition to presenting good repeatability, reversibility, and the possibility of technology transfer to flexible substrates. Furthermore, PEDOT:PSS@ZnO showed good selectivity to methanol compared to ethanol, ammonia, and CO_2_. The best devices showed responses greater than 700% in detecting methanol.

## 1 Introduction

With the rapid process of urbanization and industrial development, toxic and harmful gases including nitrogen compounds, sulfides, carbides, and volatile organic compound (VOC) gases are inevitably emitted.^1^

Methanol, a type of VOC, has applications in various industrial and commercial products, including medicines, perfumes, fuel additives, and biodiesel, but when it volatilizes into the air it has strong toxic effects on the human visual system and central nervous system. Therefore, effective and real-time detection of these harmful gases through the use of gas sensors becomes necessary.^2,3^

Many inorganic materials, such as ZnO (zinc oxide), have been used as a sensing layer for different gases.^4^ It has the advantages of biocompatibility, chemical stability, environmental compatibility, and low synthesis cost.^5−7^ However, the biggest disadvantage of these sensors is their high working temperature, which limits their application in portable devices. On the other hand, organic materials can improve gas detection performance, as well as reduce the working temperature and improve its sensitivity.^8−12^ In this context, organic conducting polymers including PEDOT:PSS have been successfully used in chemiresistive gas detection, especially for the detection of VOCs thanks to high molecular modification potentials and tunable properties.^13,14^

Gas sensors utilizing a hybrid active layer that synergistically combines the properties of inorganic and organic materials led to high response and better selectivity at low temperatures due to mutually reinforced adsorption and electronic interactions.^15−18^ Rashmi et al. studied a nanocomposite of a conjugated copolymer composed of aniline and pyrrole and ZnO that showed good detection of methanol vapors at room temperature, fast response, and good recovery on exposure to gas vapor.^19^ Seekaew et al. showed that ZnO quantum dots (QDs) decorated with carbon nanotubes (CNTs) have the highest response to methanol compared to those of other ignition times and undecorated ones, as well as high selectivity.^20^

In addition to the importance of handling appropriate materials and creating modification strategies to obtain increasingly more efficient devices, the manufacture of sensors on a large scale, through printed electronics, becomes important. This technology provides economical routes for processing various electronic materials at temperatures compatible with different types of substrates, in addition to providing simplified processing and reduced material waste.^21−24^

Here, we report the development of gas sensing devices using slot-die printing technology for the deposition of a PEDOT:PSS@ZnO hybrid active layer. We carry out morphological and chemical studies of the nanostructured active layer and the response and sensitivity of devices made of glass and PET (polyethylene terephthalate). The detection results show high sensitivity, fast response, and good selectivity to methanol.

## 2 Experimental Section

### 2.1 Materials and Preparation of Nanocomposites

In this study, nanocomposites were developed involving the conductive polymer poly(3,4-ethylenedioxythiophene)-poly(styrenesulfonate) (PEDOT:PSS), with a 1.3 wt % dispersion in H_2_O, purchased from Sigma-Aldrich, and two types of zinc oxide nanoparticles: ZnO with 30% in water, purchased from Next Chemical (named ZnO1), and ZnO with 5.6% in acetone, purchased from Infinity PV (named ZnO2). To this end, the original ZnO solutions were dissolved in water at a concentration of 1 mg/mL. The PEDOT:PSS@ZnO composites were developed in a 1:1 ratio after a previous analysis of proportions in active layers of sensors (see Figure SI.1). All solutions were subjected to mechanical agitation, followed by sonication in an Elmasonic E15H ultrasonic bath previous to their use in the slot-die printer.

### 2.2 Gas Sensor Fabrication and Characterization

Glass and PET substrates were used to manufacture the devices using interdigitated gold electrodes produced by photolithography. On the substrates, as a way to modify the surface energy and carry out cleaning, the atmospheric pressure plasma jet system (APPJ) was used with dry air. Plasma treatment was performed by using a Relyon PZ2-I Piezobrush in a dielectric barrier discharge (DBD) configuration.

For the deposition of pure materials and PEDOT:PSS@ZnO composites, the FOM Technologies slot-die printer was used on the interdigitated electrodes.

**Figure 1:**
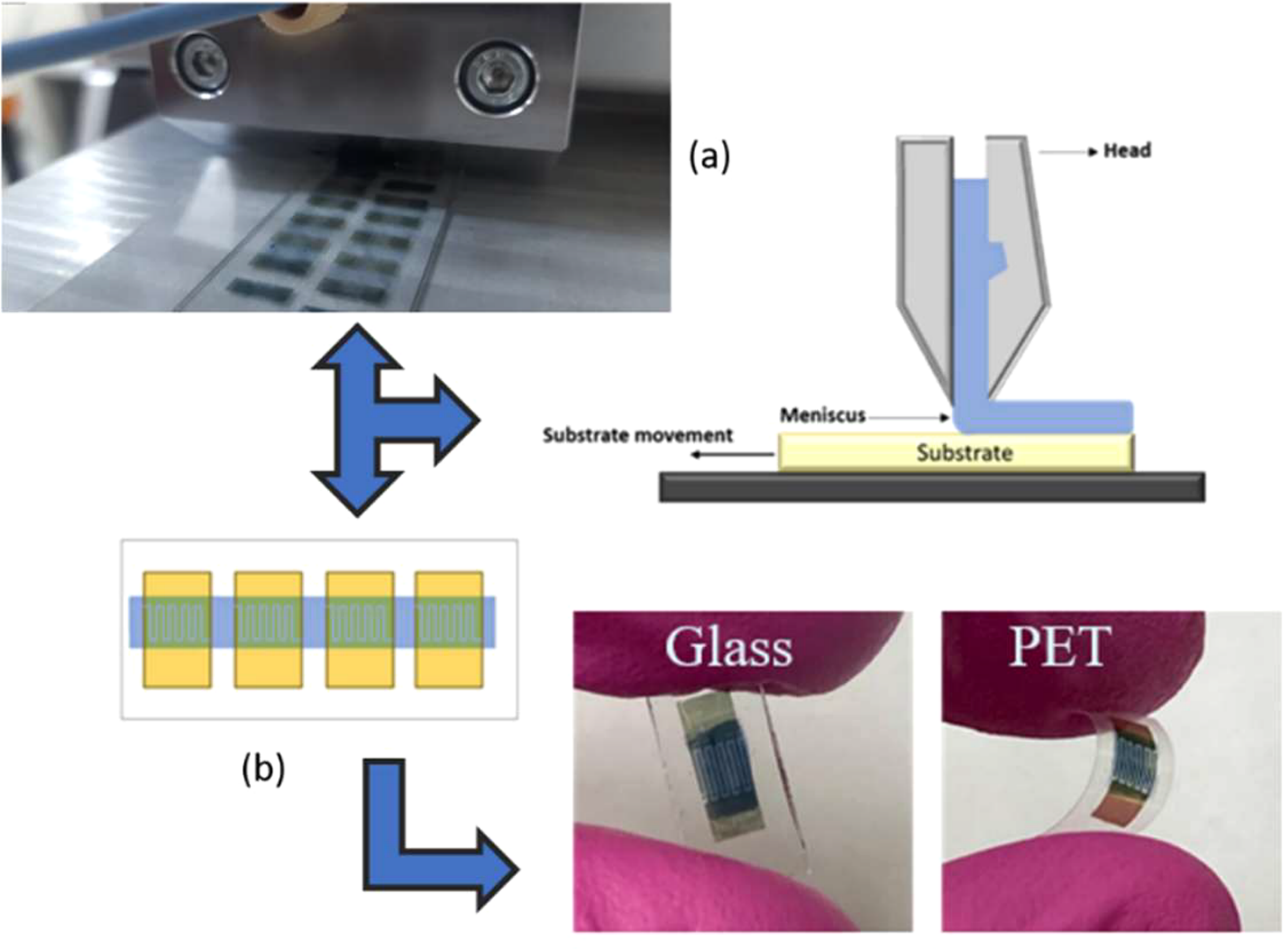
(a) Photo of the slot-die printing the devices and the schematic diagram of the functioning of the printer and (b) resulting film on interdigitated electrodes on glass and PET substrates.

In this printing process, shown in Figure 1a, the solution is pumped into the internal part of the slot-die head. Then, after following the mask, the solution is ejected through a narrow slit, forming a meniscus between the substrate and the head. When the solution is transferred from the head to the substrate (which is in motion), film formation occurs (Figure 1b). The film is formed into tracks 10 mm wide. The flow of solution injected into the slot-die head was 125 μL/min, and the speed of the deposition platform was 50 cm/min.

The measurements of the methanol detection devices were carried out in a gas sensor characterization system with solenoid valves and rotameters that control the flow of dry air and the flow of dry air plus methanol. The homemade system has cylinders with a known quantity of methanol molecules in the air, temperature sensors, and heating control.

The responses for each sensor are calculated by the relationship between the electrical resistance values in the dry air flow and the resistance values in the dry air flow plus methanol through the equation:
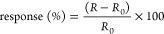
01where *R*_0_ is the resistance of the film under dry airflow and *R* is the resistance during exposure of the film to dry airflow plus methanol. The influence of the humidity and temperature was also characterized.

Morphological analyses were carried out using an atomic force microscope (AFM) in intermittent contact operation mode using a Shimadzu microscope, model SPM-9700, and also using a scanning electron microscope (SEM), FEI Quanta 450 FEG. The chemical nature of the materials was evaluated using a Witec Alpha 300R Confocal Raman Microscope, and electrical analyses were carried out using a Tektronix DMM4020 digital multimeter, an Agilent 34401A digital multimeter, and a Keysight B1500A semiconductor device analyzer.

## 3 Results and Discussion

### 3.1 Materials and Composites Properties

**Figure 2:**
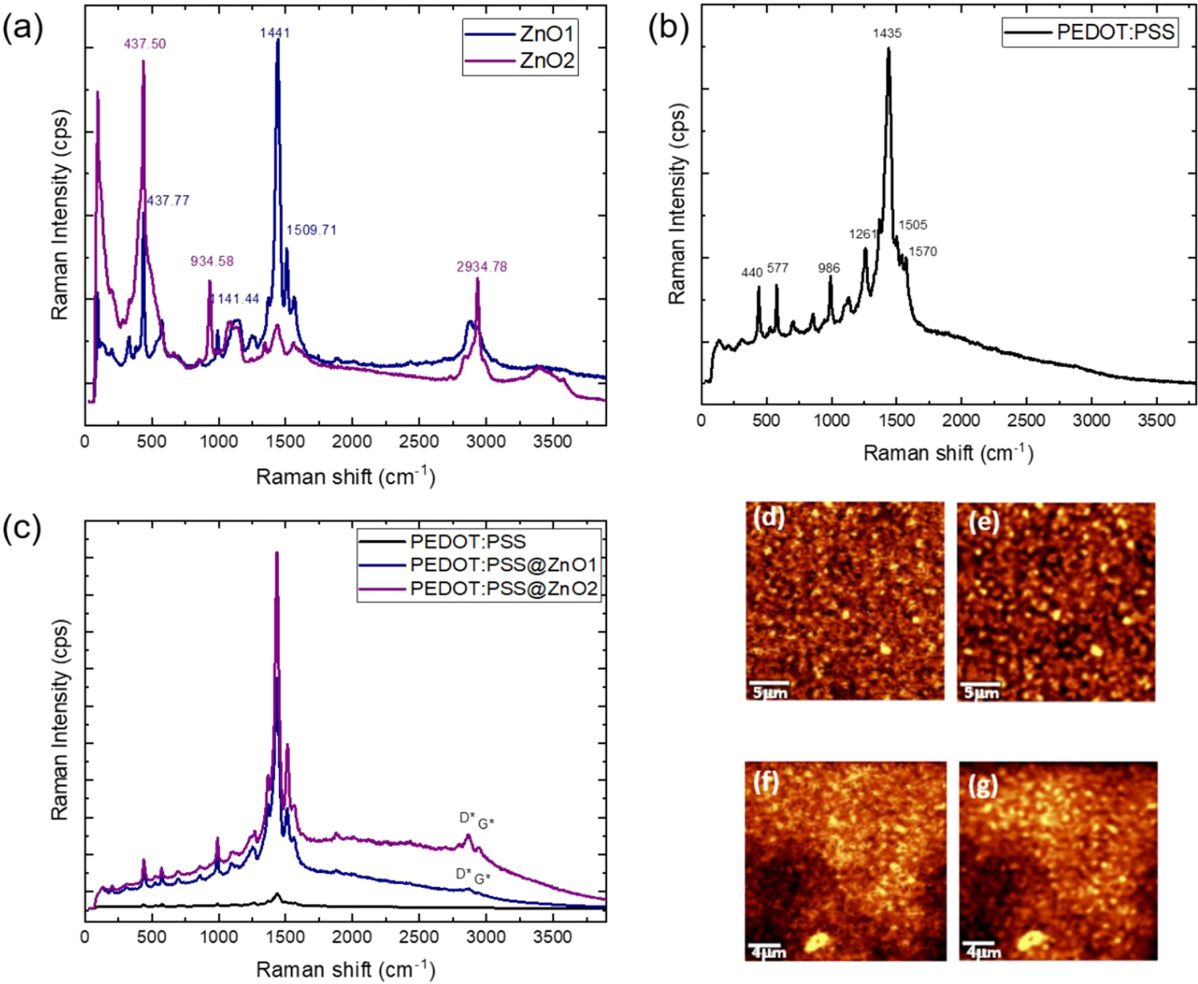
Raman spectra of (a) ZnO1 and ZnO2; (b) PEDOT:PSS; (c) PEDOT:PSS@ZnO1 and PEDOT:PSS@ZnO2. Raman mapping (d) in 440 cm^–1^ of PEDOT:PSS@ZnO1; (e) in 1435 cm^–1^ of PEDOT:PSS@ZnO1; (f) in 440 cm^–1^ of PEDOT:PSS@ZnO2; and (g) in 1435 cm^–1^ of PEDOT:PSS@ZnO2.

The Raman spectra of the neat materials and the composites are presented in Figure 2. In panel a, it is possible to compare the Raman spectra for films prepared with two different zinc oxide nanoparticles: ZnO1 and ZnO2. The Wurtzite-type ZnO crystallizes in space group *P*_6_3*mc*, featuring two formula units within its primitive cell. The optical phonons at the zone center can be categorized based on the following irreducible representation: Γ_opt_ = *A*_1_ + *E*_1_ + 2*E*_2_ + 2*B*_1_. Among these, *A*_1_ and *E*_1_ modes are polar and exhibit both Raman and infrared activity, while *E*_2_ modes (*E*_2_ low and *E*_2_ high) are nonpolar and solely Raman active and *B*_1_ modes are inactive. The prominent Raman mode at 437 cm^–1^ is predominantly attributed to the vibration of oxygen.^25^ In the Raman spectrum for ZnO1, the peaks at 1441 and 1509.71 cm^–1^ are inferred to be from the stretching vibration of the C–O and O–C–O bond originated from the acetate group, from synthesis process (Figure 2a).^25,26^

In Figure 2b, the Raman spectrum for neat PEDOT:PSS is presented, where it is possible to notice the peak at 440 cm^–1^ that corresponds to the oxyethylene ring deformation in the PEDOT:PSS structure. This spectrum exhibits two weak bands, with maxima at 1570 cm^–1^ (quinoid structure asymmetric) and 1505 cm^–1^ (C=C), and one strong band at 1435 cm^–1^ (symmetric C=C stretching vibrations).^27,28^ When the composite is studied (Figure 2c), the spectra present the properties of both materials with a predominant characteristic of PEDOT:PSS.^26,29^ Another distinctive spectral range spans from 2550 to 3100 cm^–1^, where in the second-order phonon modes manifest as G′ and D + G bands. The G′ band, occurring around 2700 cm^–1^, represents the overtone of the D band, while the D + G band, appearing at approximately 2950 cm^–1^, arises from a combination mode induced by disorder effects.^30−33^ As observed in Figure 2c, these peaks are present in both composites with different intensities and not in the pure materials. The signal for PEDOT:PSS@ZnO1 is quite small, and this observation may suggest a variance in the compound’s interaction regarding the polymer and the different zinc oxide nanoparticles ZnO1 and ZnO2. In the Raman mapping (Figure 2d–g) of the characteristic peaks corresponding to ZnO (440 cm^–1^) and PEDOT:PSS (1435 cm^–1^), it can be observed that PEDOT:PSS@ZnO2 presents a greater predominance of isolated materials on the surface of the film (Figure 2f,g), when compared to the PEDOT:PSS@ZnO1 film (Figure 2d,e). This indicates a better mixture of the polymer with ZnO1 improving the nanostructuring of the film, which can be important for the devices.

**Figure 3:**
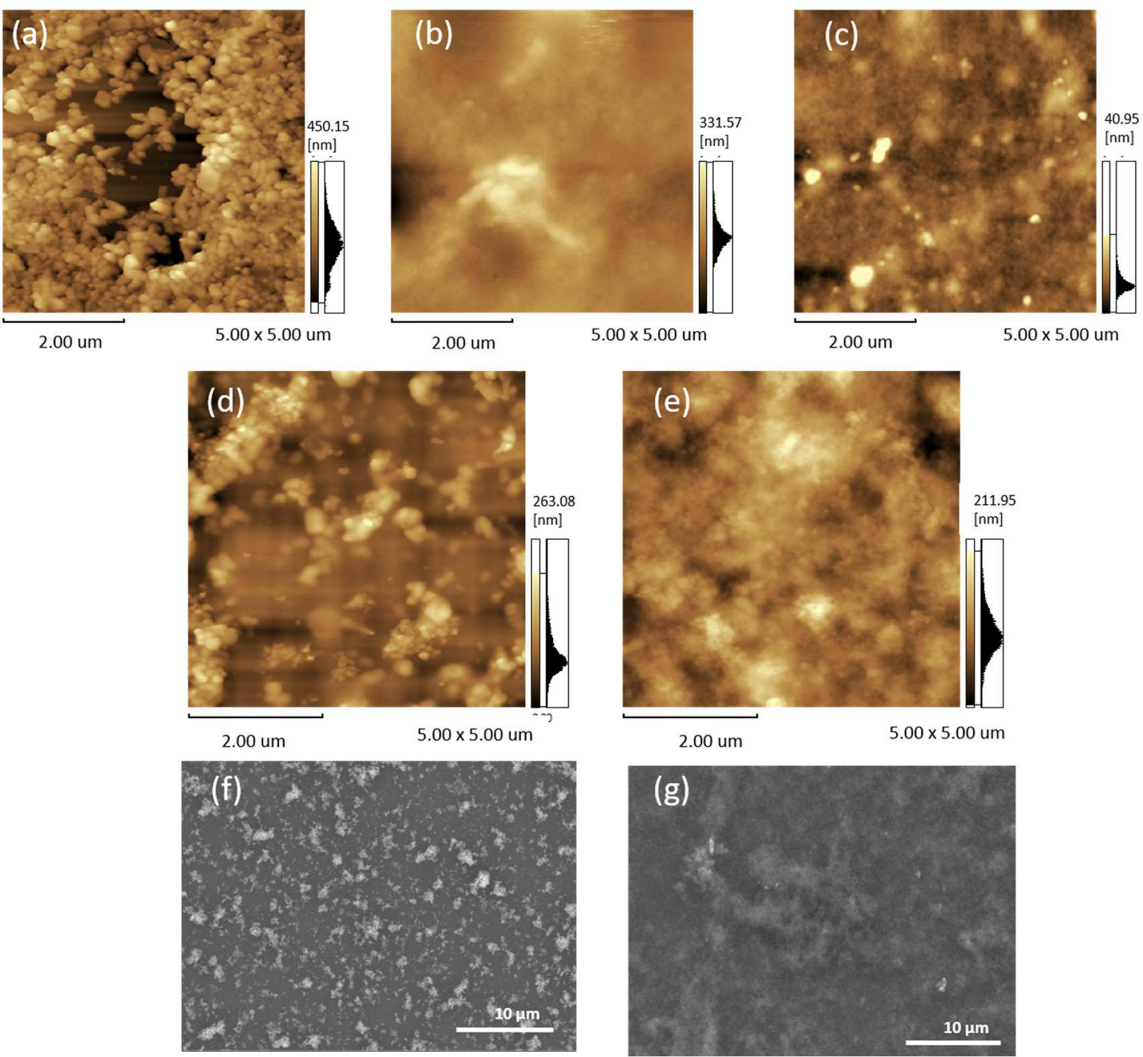
AFM images of (a) ZnO1; (b) ZnO2; (c) PEDOT:PSS; (d) PEDOT:PSS@ZnO1; and (e) PEDOT:PSS@ZnO2. SEM images of (f) PEDOT:PSS@ZnO1 and (g) PEDOT:PSS@ZnO2.

AFM images were used to record the topography of the ZnO films (Figure 3a and b) and PEDOT:PSS (Figure 3c) and PEDOT:PSS@ZnO mixtures (Figure 3d and f). Comparing the images of ZnO1 and ZnO2, it was possible to observe that ZnO1 shows a tendency to form grains with different morphologies and sizes in the range of a few tens of nanometers. Such morphology can contribute to a greater surface area of ZnO1 and consequently obtain a greater heterojunction area when mixed with PEDOT:PSS.^34^ Unlike ZnO, the pure PEDOT:PSS film exhibits a smooth and uniform morphology (Figure 3c).^35^ When mixing the two materials, mean square roughnesses of 39.2 nm for the PEDOT:PSS@ZnO1 film (Figure 3d) and 30.1 nm for PEDOT:PSS@ZnO2 demonstrate that the PEDOT:PSS@ZnO2 film is smoother than PEDOT:PSS@ZnO1 which is in agreement with the SEM images (Figure 3f and g). These studies reveal that nanostructured films can provide important properties for application in gas sensors, so that the grain size affects the resistivity of the heterojunction, making the conductive properties of the films strongly dependent on the morphological character.^36,37^

### 3.2 Gas Sensor Characteristics

Nanostructured materials were evaluated as active layers in methanol gas sensors. To achieve this, devices using printed PEDOT:PSS@ZnO nanocomposites were first subjected to dry air cycles interspersed with dry air plus 500 ppb of methanol at room temperature. The performance of the hybrid active layer was also evaluated in sensors manufactured on glass and PET substrates.

**Figure 4:**
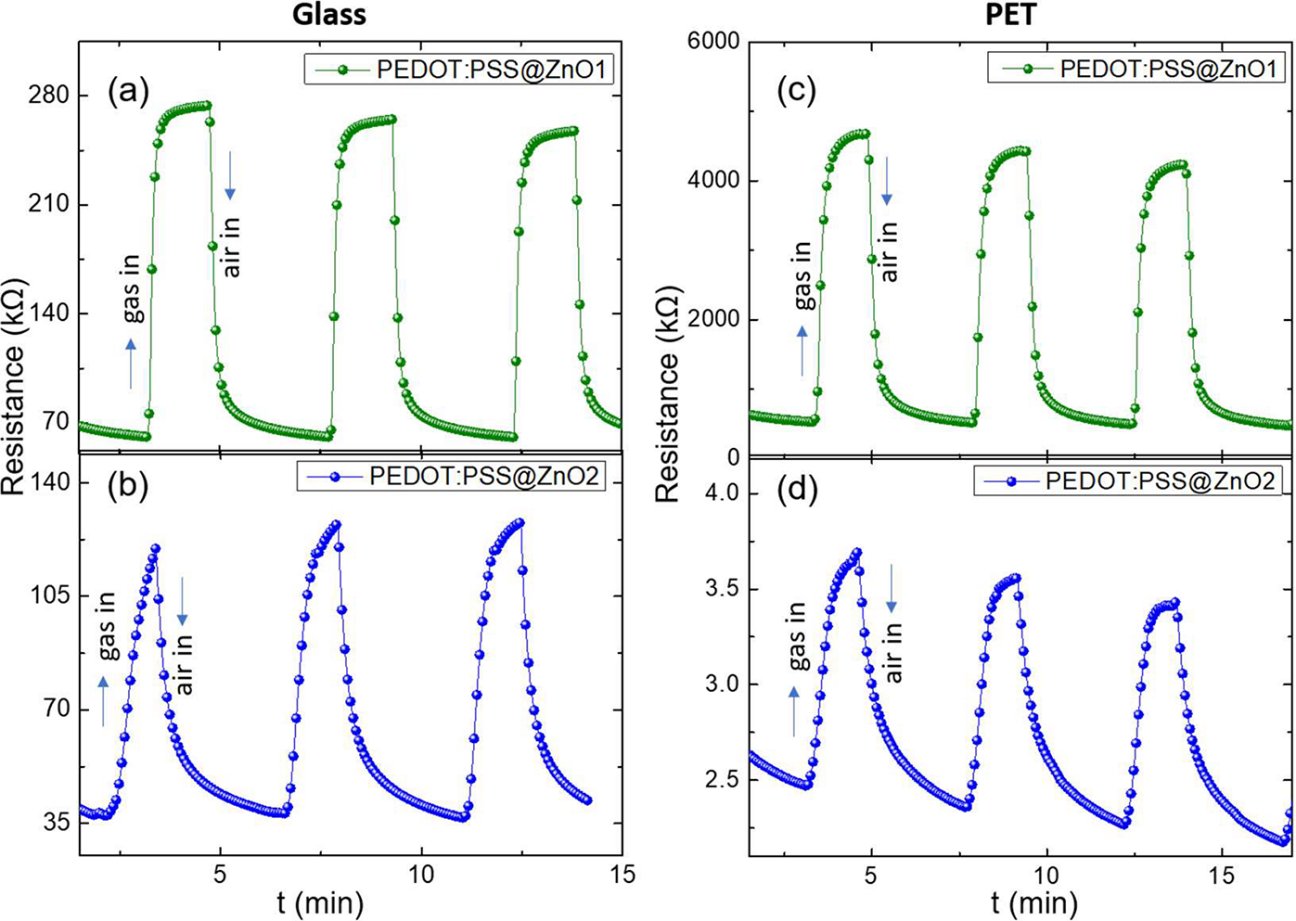
Resistance versus time for different sensing devices with the PEDOT:PSS@ZnO active layer: (a) PEDOT:PSS@ZnO1 glass; (b) PEDOT:PSS@ZnO2 glass; (c) PEDOT:PSS@ZnO1 PET; (d) PEDOT:PSS@ZnO2 PET. The measurement was done at room temperature.

Resistance versus time measurements for all devices with the PEDOT:PSS@ZnO ink are listed in Figure 4. All devices, both glass and PET, showed higher resistance in contact with methanol gas than in dry air, and all sensors showed almost the same adsorption–desorption curve with maximum and minimum responses continuously up to three cycles, which indicates that these sensors have good repeatability.^38^

**Figure 5:**
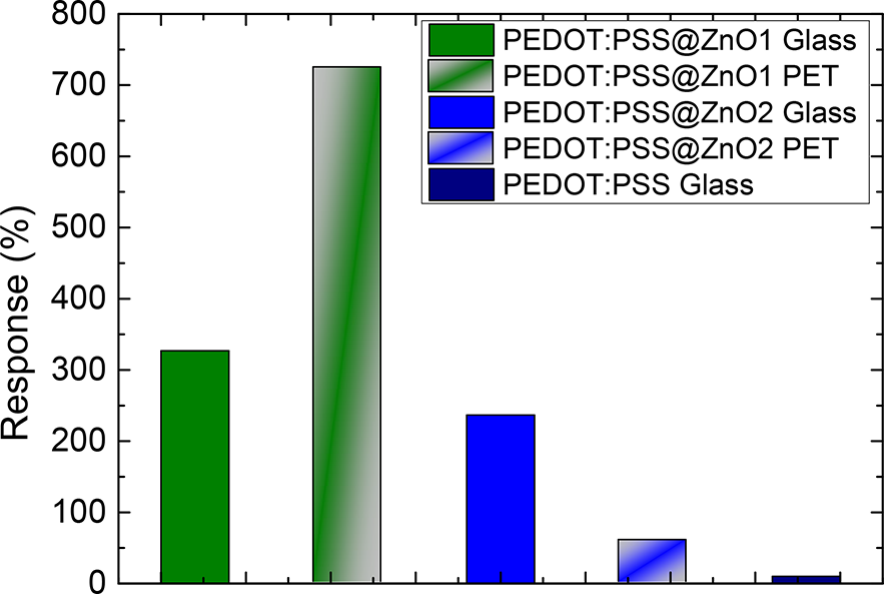
Comparison of the response of the devices. The measurement was done at room temperature.

The sensitivity results extracted from these measurements can be analyzed in Figure 5 and Table 1, and the response and recovery times are summarized in Table 1. It is possible to observe that all variations of the device showed a high response to methanol gas. Furthermore, the sensitivity presented by the device using PEDOT:PSS@ZnO1 in PET was 2.2 times greater when compared to glass, reaching a 724.7 ± 30.2% response. This result may be related to the previous treatment with an atmospheric pressure plasma jet system with natural air carried out on the substrates for manufacturing the sensors, as presented in the experimental section. Although plasma treatment has been carried out on both substrates, the literature presents several studies showing that it has been of great importance for PET substrates, improving adhesion, wettability, and printing coating processes.^39,40^ Furthermore, the roughness of the glass is greater than in PET, which may also have contributed to a lower result in the device’s performance.

**Table 1:** Values Found for Response, Response Time, and Recovery Time of PEDOT:PSS@ZnO and Pure PEDOT:PSS Devices with Methanol Detection at 500 ppb. The measurements were done at room temperature

	response (%)	*T*_res_ (min)	*T*_rec_ (min)
PEDOT:PSS@ZnO1 Glass	326.9 ± 9.9	0.41 ± 0.05	0.48 ± 0.02
PEDOT:PSS@ZnO1 PET	724.7 ± 30.2	0.53 ± 0.03	0.55 ± 0.07
PEDOT:PSS@ZnO2 Glass	236.7 ± 9.1	0.8 ± 0.1	1.0 ± 0.07
PEDOT:PSS@ZnO2 PET	60.0 ± 3.3	0.8 ± 0.02	1.0 ± 0.04
PEDOT:PSS Glass	10.1 ± 0.06	1.5 ± 0.04	1.1 ± 0.07

Comparing the devices with the PEDOT:PSS@ZnO2 layer, in glass and in PET, the response for PET was almost 75% lower when compared to glass. Since ZnO2 is suspended in acetone, the acetone may have reacted with the PET resulting in a less efficient device.

By way of comparison, we also analyzed the response of the sensor based on pure PEDOT:PSS paint with the PEDOT:PSS@ZnO composite. Here, we notice that the mixture presents a significant improvement in the response. It was also observed that the PEDOT:PSS@ZnO1 composite presented short response and recovery times, both in glass and PET, when compared to the gas sensor with pure PEDOT:PSS and PEDOT:PSS@ZnO2. Very short response and recovery times suggest faster rates of gas adsorption and desorption on the surface of sensor materials and may also be related to favorable dedoping due to the formation of a localized polaron band in the greater presence of metal oxides and greater surface roughness, which was confirmed by topographic images.^41−43^

**Figure 6:**
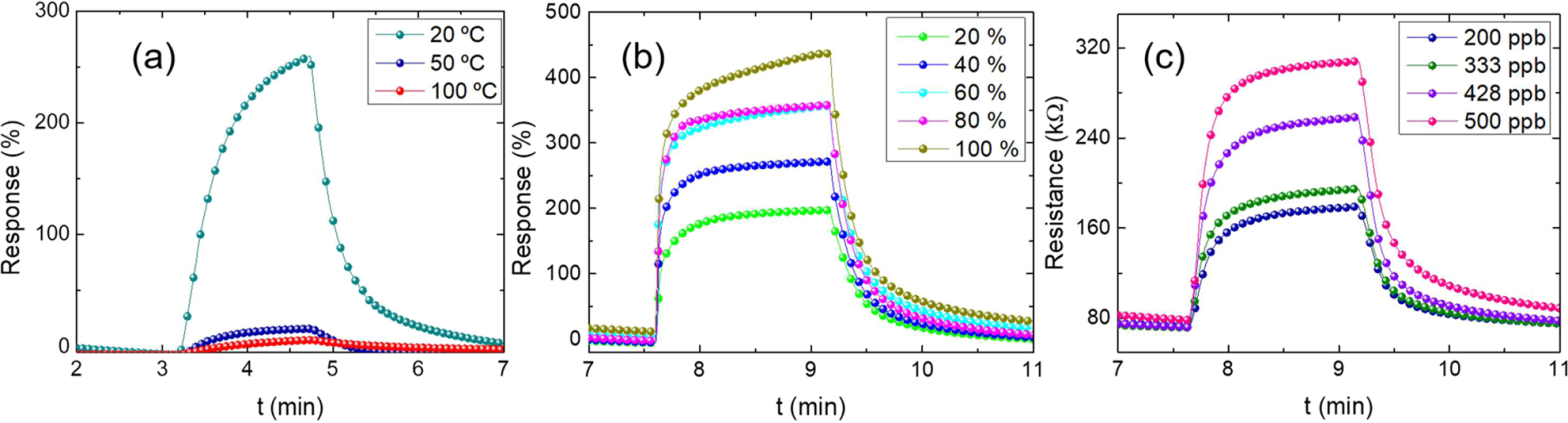
Response curves for PEDOT:PSS@ZnO1 sensors (a) for different temperatures, (b) under the influence of air humidity, and (c) for different methanol concentrations.

The study of the effect of temperature was also carried out to detect methanol gas. For this, the responses of the sensors at 20, 50, and 100 °C, on glass, were tested and are shown in Figure 6a. It was possible to observe that the absorption/desorption balance is affected with an increase in the device’s working temperature, causing a decrease in the response to methanol gas. The high sensitivity of the sensors to room temperature and the decrease in the sensors’ response to high temperatures may have occurred due to the good nanostructuring of the hybrid active layer where the use of PEDOT:PSS can reduce the working temperature of the ZnO material.^44^ Furthermore, the devices using active layer PEDOT:PSS@ZnO1 were tested in different humid environments, where the measurements were made alternating between atmospheres of 500 ppb methanol plus humid air and dry air at room temperature, and the results are shown in Figure 6b. It can be observed that the sensor response increases with increasing relative humidity (RH) value added to methanol, which can be attributed to the distortion of the nanostructure by the absorbed water molecules.^45^ It is also observed that when exposing the device to a relative humidity of 80%, a stable response is achieved. This stability may be related to the water coverage over the active layer, which leads to competitive adsorption against methanol vapor.^46^ Furthermore, the increase in sensor response with increasing humidity may be related to PEDOT:PSS. In an aqueous colloidal dispersion solution, PEDOT:PSS tends to form micellar microstructures consisting of a PEDOT-rich hydrophobic core and a PSS-rich hydrophilic shell. Therefore, PEDOT:PSS is strongly hydrophilic, and swelling of this material to the equivalent state can be achieved in seconds.^47^ Despite this characteristic, the response is lower compared with exposing the sensor to a mixture of dry air and methanol.

The sensitivity as a function of the methanol gas concentration for the PEDOT:PSS@ZnO active layer on glass is shown in Figure 6c. In this analysis, four cycles were recorded, corresponding to methanol gas concentrations of 200, 333, 428, and 500 ppb, respectively. The sensitivity of the sensors showed a good response to different concentrations of methanol even at lower concentrations.

**Figure 7:**
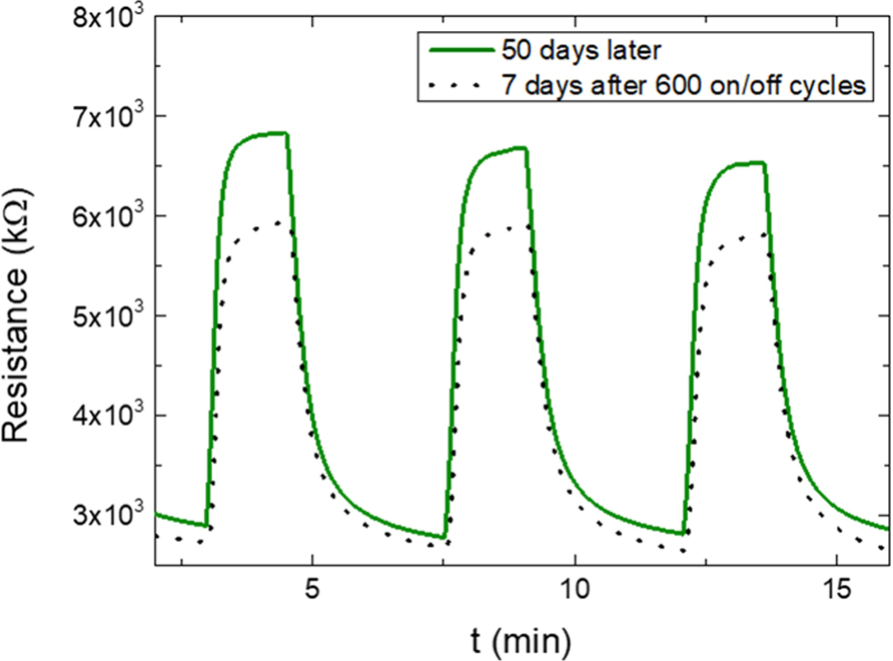
Resistance versus time for PEDOT:PSS@ZnO1 in PET with 500 ppb methanol in dry air. The purple curve is the result after 50 days of manufacturing the device, and the dashed curve is the result after 7 days of exhausting 600 on/off cycles. The measurement was done at room temperature.

After 50 days, the PET device with the PEDOT:PSS@ZnO1 active layer was again subjected to dry air cycles interspersed with dry air and 500 ppb of methanol. The device used in this measurement was stored at room temperature without being modified or treated. It was observed that the methanol sensor continued to show good repeatability. This same sensor was then exposed to exhaustive long-term measurements (Figure SI.2). For this, 600 on/off cycles were performed on three consecutive days. A decrease in the sensor response was observed at the end of each day, but at the beginning of the next day, the response returned to its initial value. This process is evidence that not all methanol molecules adsorbed are released during the cycle process at room temperature; however, after approximately 12 h, the remaining methanol molecules could be desorbed.^46^ After 7 days of the long-term experiment, it was observed that the device showed a 15% lower response compared with the beginning of the experiment, as shown in Figure 7.

**Figure 8:**
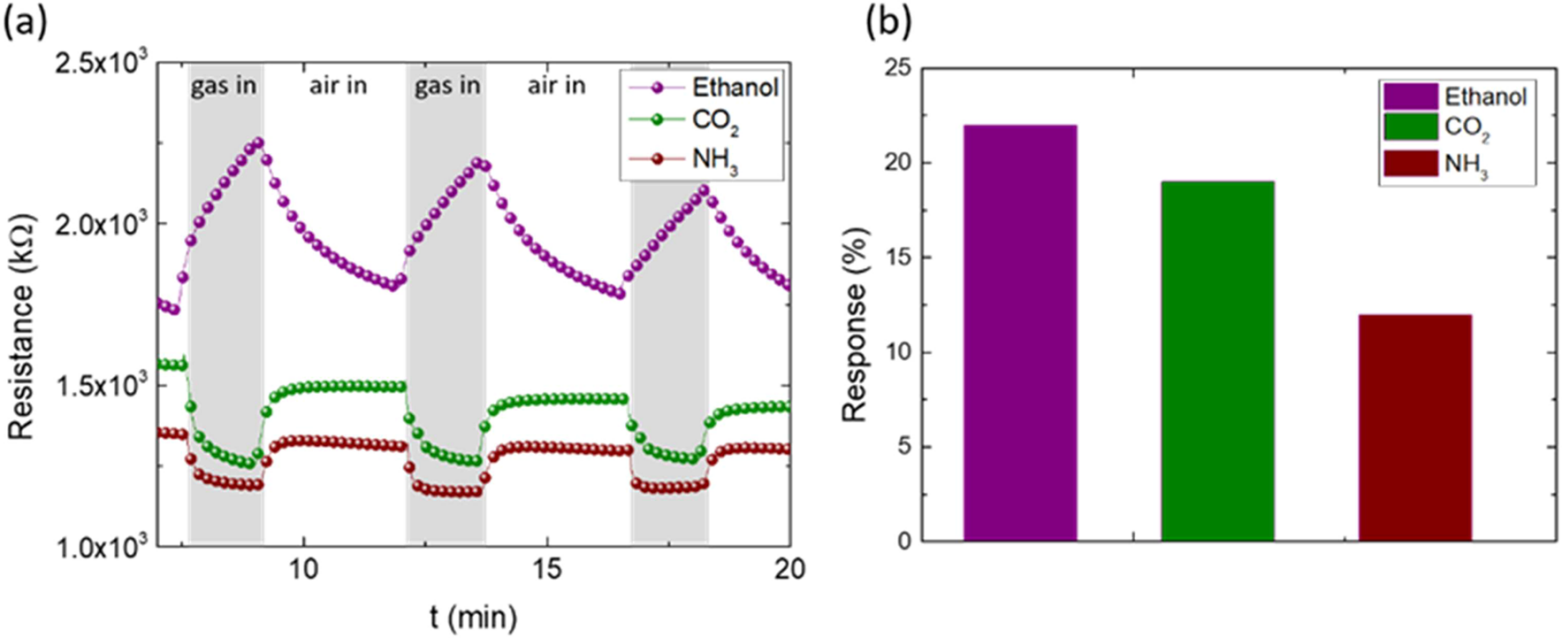
Device with the PEDOT:PSS@ZnO1 PET active layer for sensing ethanol, carbon dioxide, and ammonia: (a) resistance versus time curves and (b) comparison of responses for gases. The measurement was done at room temperature.

The study of selectivity is another important aspect of the detection performance. To this end, the PET device with the PEDOT:PSS@ZnO1 active layer was also evaluated for the gases ethanol, ammonia (NH_3_) and carbon dioxide (CO_2_), as shown in Figure 8a. It can be observed that the nanostructured material, when in contact with ethanol, presents characteristics similar to methanol but with much lower efficiency. When PEDOT:PSS@ZnO1 was exposed to NH_3_ and CO_2_ gases, the resistance variation decreases when the device is exposed to gases and the resistance increases with air intake. This result is inversely proportional to that reported for methanol gas. Furthermore, the PEDOT:PSS@ZnO1 sensors applied to ethanol, ammonia, and CO_2_ exhibited a low response value when compared to the results obtained with methanol gas, as shown in Figure 8b. This result indicates the good selectivity of the PEDOT:PSS@ZnO1 hybrid nanocomposite to methanol.

Finally, in Table 2, a comparison was made of the results analyzed in this study with those reported in the literature. As most nanomaterials that use oxides present difficulty in the sensitivity of different molecules to room temperature, the results presented here are promising, as the use of the PEDOT:PSS@ZnO hybrid nanocomposite in addition to reducing the working temperature of ZnO and consequently of the devices also demonstrated the possibility of technology transfer to flexible substrates in a roll-to-roll process.^48−50^

**Table 2:** Comparison of the Performances of Sensors for Detecting Different Gases Using Oxides in the Active Layer^a^

materials	target gases	temperature (°C)	response (%)	ref
ZnO DQs-CNTs	methanol	room temperature	15.8	(20)
CNT/ZnO	methanol	150	73	(2)
PANI@SnO2	NH3	room temperature	10	(49)
NiO-ZnO	SO2	240	16	(50)
PEDOT:PSS@ZnO	LPG	room temperature	58.8	(34)
PEDOT:PSS@ZnO1	methanol	room temperature	730	this work

a Lists of materials used, working temperature, response, and reference are provided.

## 4 Conclusion

Thin films of ZnO, PEDOT:PSS, and their compounds were successfully printed by the slot-die method for application in gas sensors. We studied two variations of ZnO for the formation of nanostructured PEDOT:PSS@ZnO composites. We were able to observe, through morphology, that the compound using PEDOT:PSS@ZnO1 presented nanograins and greater roughness when compared with the PEDOT:PSS@ZnO2 blend. These nanostructured materials were applied as an active layer in methanol gas sensors made of glass and PET. All variations presented high response values reaching 724.7 ± 30.2% for PET at room temperature. Furthermore, the devices presented good repeatability, reversibility, selectivity, low working temperature, and the possibility of technology transfer to flexible substrates that can be used in roll-to-roll processes.

## Supporting Information Available

Additional experimental details and exhaustive testing on PEDOT:PSS@ZnO1 sensor (PDF)
