# Supplementary material for: Gas Sensor Based on Highly Effective Slot-Die Printed PEDOT:PSS@ZnO Hybrid Nanocomposite for Methanol Detection
Source: ACS Appl Mater Interfaces. 2024 Jun 4;17(9):13065–73. doi: 10.1021/acsami.4c03131 (PMC11891836; doi:10.1021/acsami.4c03131)
Supplement: Supplementary file 1 — am4c03131_si_001.pdf [file am4c03131_si_001.pdf]

Supporting Information

Gas sensor based on highly effective slot-die printed PEDOT:PSS@ZnO hybrid nanocomposite for methanol detection

Talitha Ramos Canabarra dos Santos[a], Maiara de Jesus Bassi[a,c*], Morgana Muller de França[b], Júlia Ketzer Majewski[a,c], Marcos Vinícius Woiski Barcote[a,c], Anne Elize Puppi Stanislawczuk[c], Lucimara Stolz Roman[a,b *].

[a] Nanostructured Devices Laboratory at Physics Department, Federal University of Paraná, 81531-980, Curitiba, Brazil.

[b] PIPE- Graduate Program in Materials Science and Engineering of Federal University of Paraná, 81531-980, Curitiba, Brazil.

[c] Next Chemical, João Chede, 2245, 81170-220, Curitiba, Brazil.

* Corresponding authors: Lucimara.roman@ufpr.br and maibassi@ufpr.br.

Experimental details:

The ratio 1:1 for PEDOT:PSS@ZnO was chosen for further study due to the response on different concentrations. PEDOT:PSS on the blend. The best devices considering both ZnO nanoparticles were achieved for 50%. Neat ZnO devices have no response for methanol vapor.

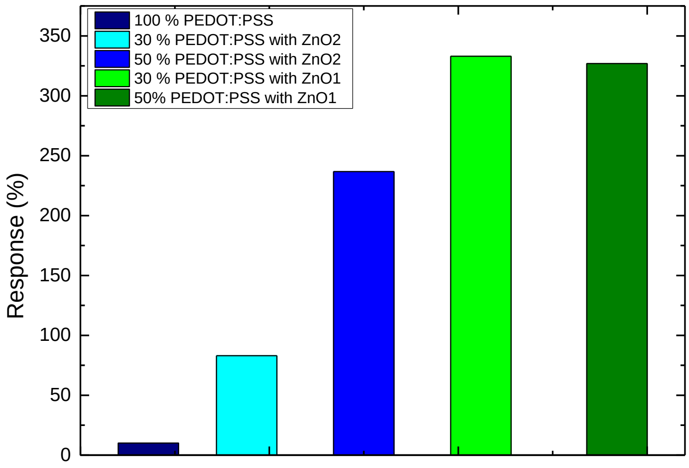

Figure S1: Response of devices to various concentrations.

Details regarding the photolithography used on gold electrodes.

Photolithography consists of the following steps: (a) Deposition of the photoresistive material with subsequent spreading by spin coating; (b) Pre-baking the glass substrate with photoresistive material for 10 minutes at 80 °C; (c) Exposure of the material to UV light (10 mW/cm2) for 10 seconds using an acetate mask with the interdigitated mold, (d) Bath revealing the photoresistive material in xylene for 20 seconds followed by cleaning with isopropyl alcohol and thermal treatment at 120 °C, (e) metallization with gold on the glass and PET substrates and (f) Removal of the photoresistive material not exposed to ultraviolet light together with the metals superimposed on these regions, with a hot chloroform bath, plus 10% in volume of remover, under mechanical agitation.

Sensor's reproducibility tests:

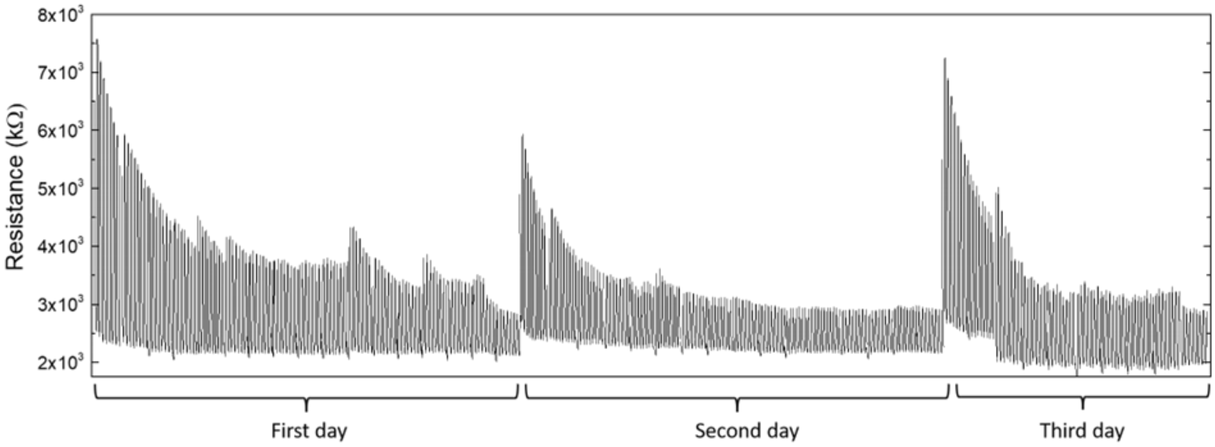

Figure S2: Exhaustive test on our 50 day old sensor - Resistance measurements for the PEDOT:PSS@ZnO device on PET for 3 consecutive days and 600 on/off cycles.
